# Association of serum calcium levels within the first hour of ER admission with 28-day mortality in sepsis: A retrospective ICU analysis

**DOI:** 10.1097/MD.0000000000046699

**Published:** 2025-12-19

**Authors:** Lijun Xu, Haoran Peng, Lijie Qin, Yanwei Cheng

**Affiliations:** a Department of Emergency, Henan Provincial People’s Hospital, People’s Hospital of Zhengzhou University, People’s Hospital of Henan University, Zhengzhou, China.

**Keywords:** 28-day mortality, prognostic marker, retrospective analysis, sepsis prognosis, serum calcium levels

## Abstract

Sepsis poses a global health challenge, necessitating effective predictors for risk stratification and timely intervention. Serum calcium, involved in vital physiological processes, has shown potential associations with sepsis outcomes, but the evidence remains limited and conflicting. This retrospective study included 1000 sepsis patients admitted to the intensive care unit. Serum calcium levels were measured within 1 hour of emergency room (ER) admission. Clinical data, including demographics, comorbidities, vital signs, laboratory results, and severity scores, were collected. Logistic regression and receiver operating characteristic curve analyses were conducted to assess the association between serum calcium levels and 28-day mortality. Kaplan–Meier analysis assessed the effect of serum calcium on 28-day survival. Lower serum calcium levels within 1 hour of ER admission were significantly associated with higher 28-day mortality (adjusted odds ratio: 0.1097, 95% CI: 0.04024–0.2912, *P* < .0001), even after adjusting for confounders. The area under the curve for serum calcium in predicting 28-day mortality was 0.5885 (*P* < .0001). Combining serum calcium with other predictors, such as the Charlson comorbidity index, red cell distribution width, lactic acid levels, and Acute Physiology and Chronic Health Evaluation II score, improved predictive accuracy (area under the curve: 0.7245, *P* < .0001). Kaplan–Meier analysis demonstrated a significant association between lower serum calcium levels and reduced 28-day survival (*P* < .0001). Low serum calcium levels within 1 hour of ER admission are significantly associated with increased 28-day mortality in sepsis patients. It complements existing predictors, showcasing its potential role in a comprehensive prognostic framework.

## 1. Introduction

Sepsis, characterized by life-threatening multiorgan dysfunction caused by a dysregulated host response to infection, remains a critical global health challenge.^[1]^ Annually, sepsis affects an estimated 49 million individuals, culminating in over 11 million fatalities.^[2]^ A consequential fraction of survivors manifests persistent functional limitations.^[3]^ Despite remarkable medical advancements, there remains an exigent need to unearth steadfast markers predictive of sepsis outcomes, thus enabling judicious therapeutic interventions.

Extensive research has explored various biomarkers and clinical indicators as potential predictors of sepsis mortality, including well-studied inflammatory markers like procalcitonin (PCT) and C-reactive protein (CRP), as well as established organ dysfunction scores such as Acute Physiology and Chronic Health Evaluation II (APACHE II) score and lactic acid (Lac) levels.^[4–8]^ However, the complex and heterogeneous nature of sepsis has made it difficult to reliably predict patient outcomes using these measures.

Recent attention has turned to serum calcium, a vital mineral involved in multiple physiological processes, including cell signaling, muscle contraction, coagulation, and immune regulation.^[9–11]^ Disturbances in calcium homeostasis have been widely observed in sepsis patients, suggesting a possible association with clinical outcomes.^[12]^ Several investigations have reported that hypocalcemia is linked to higher mortality risk in critically ill or septic patients, while others have observed less consistent associations, highlighting the heterogeneity of patient populations and study designs.^[13,14]^ Moreover, inappropriate correction of hypocalcemia might carry risks in critically ill patients.^[15]^ Experimental and mechanistic studies further suggest that calcium dysregulation during sepsis may stem from blood-to-tissue redistribution and subcellular calcium overload, emphasizing the complex pathophysiological nature of calcium disturbances.^[16]^

Therefore, the primary objective of this single-center retrospective study is to assess the association between serum calcium levels within 1 hour of emergency room (ER) admission and 28-day mortality in sepsis patients, leveraging an expansive cohort. By addressing this knowledge gap, we aim to provide valuable insights into the predictive value of serum calcium in sepsis, thereby facilitating risk stratification and optimizing patient management. The significance of this study lies in its use of a substantial sample size, allowing for more conclusive evaluations of the association between serum calcium levels and sepsis mortality.

## 2. Materials and methods

### 2.1. Study design and patient cohort

This retrospective single-center observational study was executed within the intensive care unit (ICU) of Henan Provincial People’s Hospital spanning January 2019 through July 2023. Ethical approval was obtained from the hospital’s ethics committee (approval number: 2019057), and written informed consent was obtained from all participants or their guardians.

Inclusion criteria focused on individuals admitted to the ICU via our ER diagnosed with sepsis, adjudicated by the “sepsis-3” consensus definition.^[1]^ Exclusions were applied to individuals under 18 years or over 80 years of age, those transferred from other hospitals, and patients who died within 24 hours of ICU admission, as well as pregnant or lactating individuals. Additionally, those with preexisting conditions affecting serum calcium levels (e.g., chronic renal failure, parathyroid disorders, acute pancreatitis, malignancies, other bone metabolism disorders), those who had recently used immunosuppressants, calcium, vitamin D preparations, or other drugs that could influence electrolyte levels within the preceding 3 months were excluded. Moreover, patients with missing data on serum calcium measured within 1 hour of ER admission, those receiving treatment prior to laboratory testing, and those lost to follow-up at 28 days were also excluded from the study. The analysis also excluded patients whose family members withheld aggressive treatment.

### 2.2. Data collection

Clinical data were collected from electronic medical records for all enrolled sepsis patients. This comprehensive dataset included essential demographics such as age, gender, smoking, and alcohol abuse, as well as the Charlson comorbidity index (CCI) and vital signs upon presentation in the ER including heart rate, respiratory rate (RR) and mean blood pressure. Laboratory data measured within 1 hour of ER admission encompassed white blood cell count (WBC), blood platelet count (PLT), hemoglobin, red cell distribution width (RDW), serum creatinine (Scr), blood urea nitrogen (BUN), serum potassium, serum calcium, and arterial PH, Lac. Additional information comprised the sequential organ failure assessment (SOFA) score, APACHE II score, the primary infection site, the occurrence of mechanical ventilation, the duration of mechanical ventilation, and the length of stay (LOS) in the ICU. Patients were followed up until death or for 28 days after ICU admission. Based on their survival status at the 28-day follow-up, sepsis patients were further classified into 2 groups: survivors and non-survivors.

### 2.3. Statistical analysis

Continuous variables were presented as either mean ± standard deviation or as median with interquartile range, contingent upon data normality assessed using Kolmogorov–Smirnov test. Categorical variables were present as counts and proportions. For continuous variables, either Student *t* test or the Mann–Whitney *U* test was applied, while categorical variables were assessed using either the Chi-square test or Fisher exact test. Additionally, multivariate logistic regression analysis was performed to adjust for potential confounding variables identified in univariate logistic regression analysis with a *P* value < .10. Receiver operating characteristic (ROC) curve analysis was employed to determine the predictive accuracy of serum calcium in predicting sepsis-related 28-day mortality, with calculation of the area under the curve (AUC) along with odds ratio (OR) and 95% confidence interval (CI). To visualize cumulative survival, Kaplan–Meier curves were constructed, and differences in cumulative survival between high and low serum calcium level groups were assessed using the Log-rank test. All statistical analyses were conducted using GraphPad Prism 9.0 software (GraphPad Software Inc., San Diego), with a significance threshold set at *P* value < .05.

## 3. Results

### 3.1. Baseline and clinical characteristics of the study population

**Figure 1.**
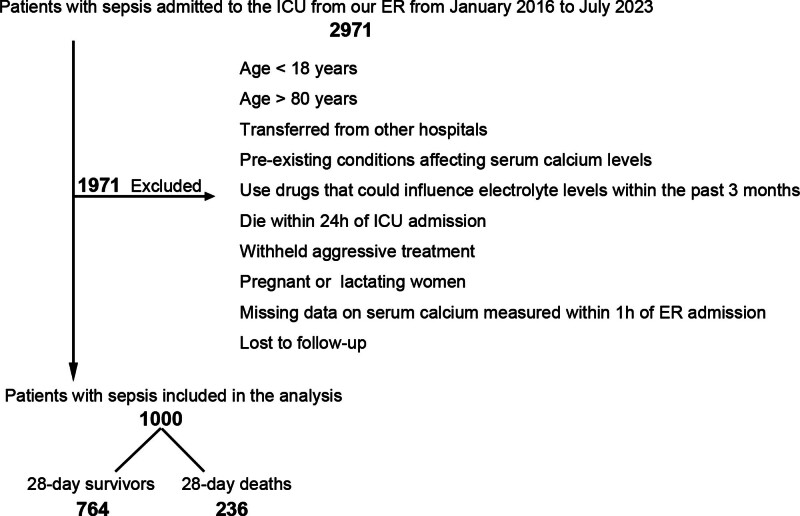
Flow chart for the process of inclusion steps and exclusion steps.

A total of 2971 sepsis patients who were admitted to the ICU from our ER between 2016 and 2023 underwent initial screening. Among them, 1971 patients were subsequently excluded, resulting in a final cohort of 1000 individuals who were included in this study (Fig. 1).

**Figure 2.**
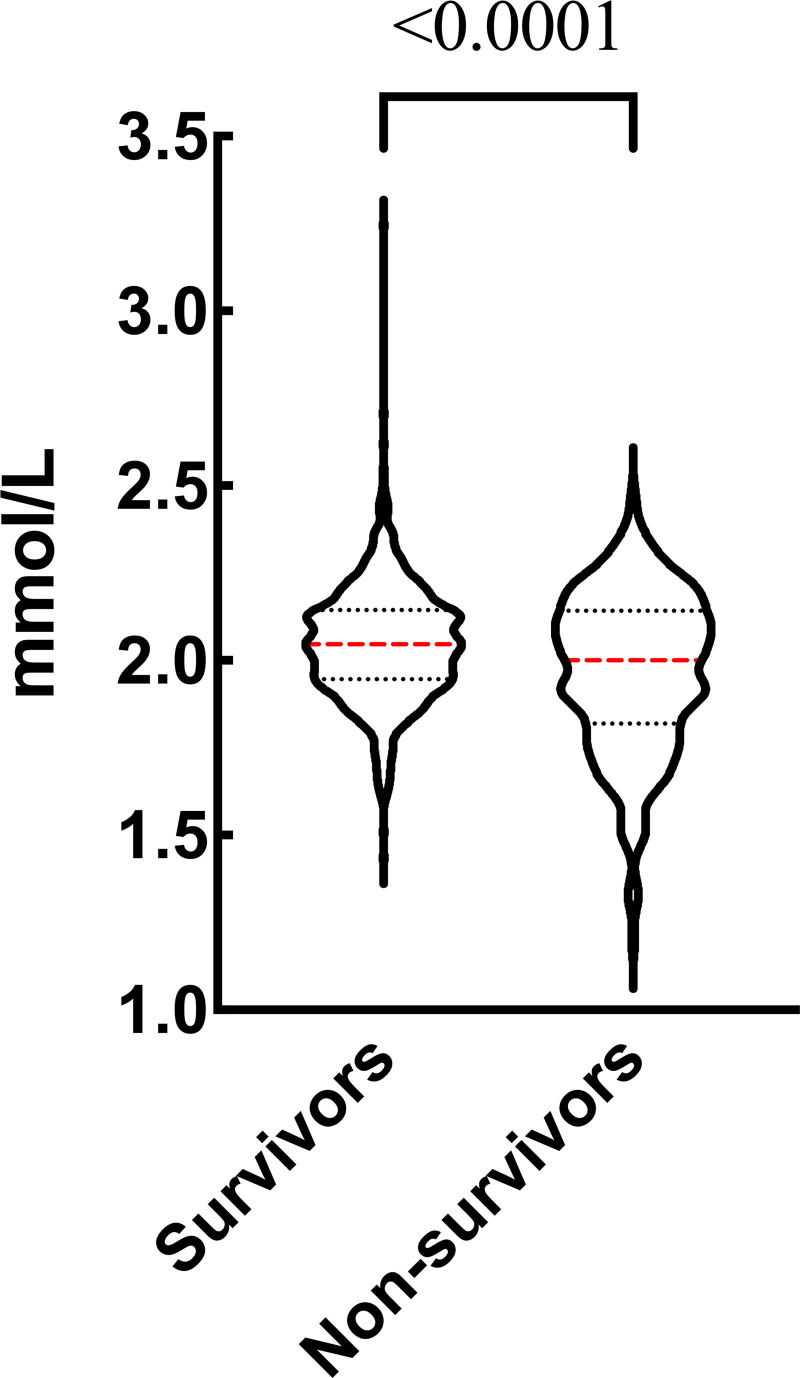
Serum calcium levels within 1 hour of ER admission in survivors and non-survivors. ER = emergency room.

Table 1 outlines the detailed baseline and clinical characteristics. Of the cohort, 680 (68.27%) were male and the median age was 59 years. The median APACHE II score and SOFA score were 17 and 5, respectively. The 3 most frequently encountered primary infection sites were abdominal (28.8%), respiratory (19.1%), and urinary (14.7%). Notably, 434 (43.4%) patients underwent mechanical ventilation, with a median duration of ventilation of 21 hours. The median LOS in ICU was 4 days. Total of 236 patients (23.6%) died during the 28-day follow-up period. Compared with survivors, non-survivors exhibited advanced age (median 62 vs 58, *P* = .002) and a higher CCI (median 4 vs 3, *P* < .0001). Additionally, non-survivors displayed an increased RR (median 20 vs 18, *P* < .0001), elevated APACHE II scores (median 20 vs 16, *P* < .0001), higher SOFA scores (median 7 vs 5, *P* < .0001), and an extended duration of mechanical ventilation (median 20 vs 16, *P* < .0001). Moreover, non-survivors exhibited elevated levels of WBC, RDW, Scr, BUN, and Lac, while lower serum calcium levels and arterial pH (*P* < .0001) (Fig. 2). No significant differences were noted in terms of gender, mean blood pressure, hemoglobin, platelet count, serum potassium, primary site of infection, and LOS in ICU.

**Table 1:** Baseline and clinical characteristics of the study population.

Variables	Total (n = 1000)	Survivors (n = 764)	Non-survivors (n = 236)	*P*-value
Demographics				
Age (yr), median (IQR)	59 (46, 68)	58 (45, 67)	62 (50, 71)	.0020
Male, n (%)	680 (68.27)	526 (68.85)	154 (65.25)	.3009
Smoking, n (%)	247 (24.81)	200 (26.18)	47 (19.92)	.0512
Alcohol abuse, n (%)	141 (12.63)	111 (14.53)	40 (16.95)	.3640
CCI, median (IQR)	3 (2, 4.75)	3 (2, 4)	4 (2, 5)	<.0001
Vital signs				
HR (beats/min), median (IQR)	87 (76, 102)	87 (77, 102)	87 (73, 101)	.4422
RR (beats/min), median (IQR)	18 (15, 23)	18 (15, 22)	20 (16, 24)	.0089
MBP (mm Hg), median (IQR)	80 (69, 92)	79 (70, 92)	83 (68, 92)	.4408
Laboratory tests				
WBC (10^9^/L), median (IQR)	15.6 (12.2, 19.6)	15.4 (12.2, 19.3)	16.1 (12.3, 21.2)	.0204
Hb (g/L), mean ± SD	110.3 ± 20.6	109.4 ± 19.1	113.4 ± 24.5.5	.0517
PLT (10^9^/L), median (IQR)	187 (142, 233)	185 (140, 231)	191 (149, 241)	.1096
RDW (%), median (IQR)	13.8 (13.2, 14.7)	13.7 (13.1, 14.5)	14.2 (13.4, 15.3)	<.0001
Scr (µmol/L), median (IQR)	88.4 (70.7, 106.1)	88.4 (70.7, 106.1)	97.2 (70.7, 123.8)	.0004
BUN (mmol/L), median (IQR)	6.1 (4.6, 8.2)	5.7 (4.6, 7.8)	6.8 (5.0, 9.5)	<.0001
Potassium (mmol/L), median (IQR)	4.1 (3.8, 4.4)	4.1 (3.8, 4.4)	4.1 (3.8, 4.5)	.1844
Calcium (mmol/L), median (IQR)	2.0 (1.9, 2.1)	2.0 (1.9, 2.1)	2.0 (1.8, 2.1)	<.0001
PH, median (IQR)	7.38 (7.31, 7.42)	7.38 (7.32, 7.42)	7.36 (7.27, 7.42)	<.0001
Lac (mmol/L), median (IQR)	1.6 (1.1, 2.4)	1.5 (1.1, 2.3)	2.0 (1.3, 3.5)	<.0001
Primary infection site				
Respiratory infection, n (%)	191 (19.1)	143 (18.7)	48 (19.8)	.7198
Abdominal infection, n (%)	288 (28.8)	229 (30.0)	59 (25.0)	.1402
Urinary infection, n (%)	147 (14.7)	105 (13.7)	42 (17.8)	.1243
Skin and soft tissue infections, n (%)	124 (1.24)	102 (13.4)	22 (9.3)	.1007
Bloodstream infection	123 (12.3)	87 (11.8)	36 (15.5)	.1469
Other/unknown, n (%)	127 (127.0)	98 (13.3)	29 (11.0)	.3293
APACHE II score, median (IQR)	17 (13, 22)	16 (12, 20)	20 (16, 26)	<.0001
SOFA score, median (IQR)	5 (4, 8)	5 (4, 7)	7 (5, 9)	<.0001
Ventilator use, n (%)	434 (43.4)	256 (33,5)	236 (100)	<.0001
Duration of mechanical ventilation (h)	21 (6, 68)	16 (5, 52)	20 (51, 96)	<.0001
LOS in ICU, days, median (IQR)	4 (2, 9)	4 (2, 9)	4 (3, 8)	.1226

APACHE II = Acute Physiology and Chronic Health Evaluation II, BUN = blood urea nitrogen, CCI = Charlson comorbidity index, Hb = hemoglobin, HR = heart rate, ICU = intensive care unit, IQR = interquartile range, Lac = lactic acid, LOS = the length of stay, MBP = mean blood pressure, PLT = blood platelet count, RDW = red cell distribution width, RR = respiratory rate, Scr = serum creatinine, SD = standard deviation, SOFA = sequential organ failure assessment, WBC = white blood cell count.

### 3.2. Serum calcium is an independent risk factor for 28-day mortality in sepsis

To explore whether a low serum calcium level within 1 hour of ER admission could be a risk factor for 28-day mortality in sepsis, logistic regression analysis was conducted. Our findings revealed that a significant association between serum calcium levels (OR: 0.1, 95% CI: 0.04280–0.2274, *P* < .0001) and 28-day mortality among sepsis patients in the univariate logistic regression model (Table 2). Even after adjusting for various confounders including age, smoking, CCI, RR, WBC, RDW, Scr, BUN, arterial pH, Lac, APACHE II score, and SOFA score, serum calcium levels within 1 hour of ER admission (OR: 0.1097, 95% CI: 0.04024–0.2912, *P* < .0001) remained an independent predictor for 28-day mortality (Table 2). Other independent factors including CCI (OR: 1.306, 95% CI: 1.139–1.498, *P* = .001), RDW (OR: 1.309, 95% CI: 1.165–1.473, *P* < .0001), Lac (OR: 1.169, 95% CI: 1.059–1.296, *P* < .0001), APACHE II score (OR: 1.072, 95% CI: 1.039–1.105, *P* < .0001) were also significantly associated with 28-day mortality (Table 2).

**Table 2:** Logistic regression models of factors related to 28-day mortality in sepsis patients.

Variables	Univariate regression mode			Multivariate regression model		
	OR	95% CI	*P* value	OR	95% CI	*P* value
Age	1.015	1.006–1.026	.0022			
Smoking	0.7013	0.4862–0.9959	.052			
CCI	1.196	1.110–1.289	<.0001	1.306	1.139–1.498	.0001
RR	1.042	1.017–1.068	.001			
WBC	1.021	1.003–1.040	.027			
RDW	1.278	1.165–1.403	<.0001	1.309	1.165–1.473	<.0001
Scr	1.003	1.001–1.005	0.0011			
BUN	1.055	1.026–1.084	.0001			
Calcium	0.09957	0.04280–0.2274	<.0001	0.1097	0.04024–0.2912	<.0001
PH	0.03475	0.009163–0.1270	<.0001			
Lactic acid	1.302	1.196–1.425	<.0001	1.169	1.059–1.296	<.0001
APACHE II score	1.094	1.070–1.118	<.0001	1.072	1.039–1.105	<.0001
SOFA score	1.173	1.115–1.234	<.0001			

APACHE II = Acute Physiology and Chronic Health Evaluation II, BUN = blood urea nitrogen, CCI = Charlson comorbidity index, CI = confidence interval, Lac = lactic acid, OR = odds ratio, RDW = red cell distribution width, RR = respiratory rate, Scr = serum creatinine, SOFA = sequential organ failure assessment, WBC = white blood cell count.

### 3.3. Predictive value of serum calcium for 28-day mortality in sepsis

**Figure 3.**
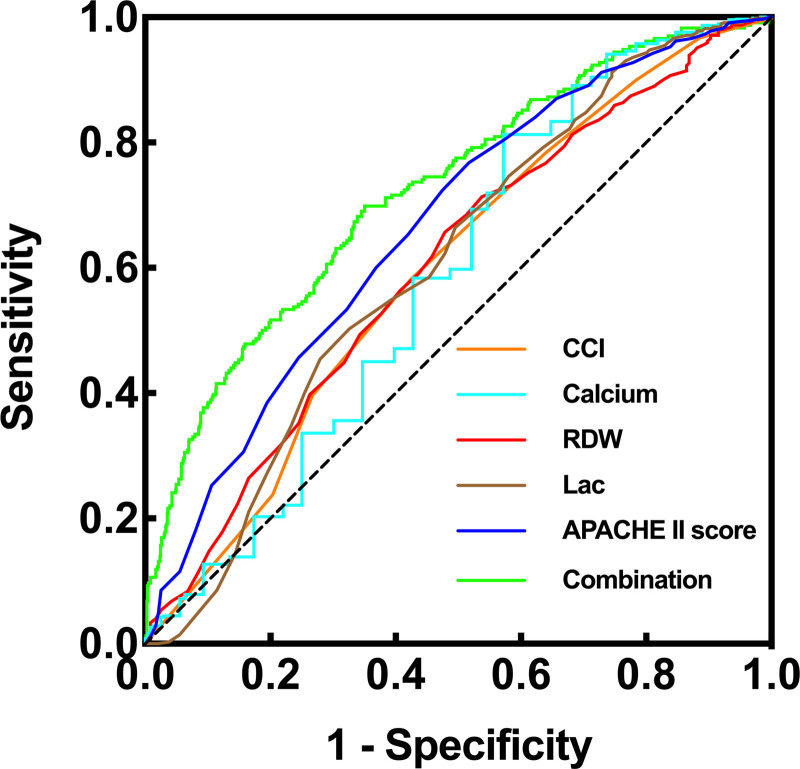
The predictive value of serum calcium levels within 1 hour of ER admission for 28-day mortality in sepsis patients. ER = emergency room.

The analysis of the ROC curve yielded an AUC of 0.5885 (95% CI: 0.5428–0.6343, *P* < .0001) for serum calcium within 1 hour of ER admission in predicting 28-day mortality risk (Table 3 and Fig. 3). Using a cutoff value of 1.91 mmol/L for predicting 28-day mortality risk, the sensitivity was 0.8128 (95% CI: 0.7836–0.8389), and the specificity was 0.3517 (95% CI: 0.2936–0.4145). Comparable predictive capacities were observed for CCI (AUC: 0.597, 95% CI: 0.5538–0.6403, *P* < .0001), RDW (AUC: 0.6032, 95% CI: 0.5613–0.6451, *P* < .0001), Lac (AUC: 0.6065, 95% CI: 0.5623–0.6507, *P* < .0001), and APACHE II score (AUC: 0.666, 95% CI: 0.6256–0.7064, *P* < .0001) with respect to 28-day mortality risk prediction. The respective cutoff values alongside their corresponding sensitivity and specificity are delineated in Table 3. Notably, the combined effect of these factors exhibited a robust predictive capacity (AUC: 0.7245, 95% CI: 0.6871–0.7618, *P* < .0001) for 28-day mortality risk in sepsis (Table 3 and Fig. 3).

**Table 3:** ROC curve analysis of predicting 28-day mortality in sepsis patients.

Variables	AUC	*P*-value	95% CI	Cutoff value	Sensitivity(95% CI)	Specificity (95% CI)
CCI	0.597	<.0001	0.5538–0.6403	3.5	0.5812 (0.5458–0.6156)	0.5763 (0.5125–0.6376)
RDW	0.6032	<.0001	0.5613–0.6451	14.15	0.6571 (0.6227–0.6899)	0.5212 (0.4576–0.5841)
Calcium	0.5885	<.0001	0.5428–0.6343	1.91	0.8128 (0.7836–0.8389)	0.3517 (0.2936–0.4145)
Lac	0.6065	<.0001	0.5623–0.6507	1.55	0.5026 (0.4672–0.5380)	0.6737 (0.6115–0.7303)
APACHE II score	0.666	<.0001	0.6256–0.7064	20.5	0.7683 (0.7371–0.7969)	0.4831 (0.4201–0.5466)
Combination	0.7245	<.0001	0.6871–0.7618			
CCI	0.597	<.0001	0.5538–0.6403	3.5	0.5812 (0.5458–0.6156)	0.5763 (0.5125–0.6376)

APACHE II = Acute Physiology and Chronic Health Evaluation II, AUC = area under the curve, CCI = Charlson comorbidity index, RDW = red cell distribution width, ROC = receiver operating characteristic.

### 3.4. Serum calcium and accumulating 28-day survival in sepsis

**Figure 4.**
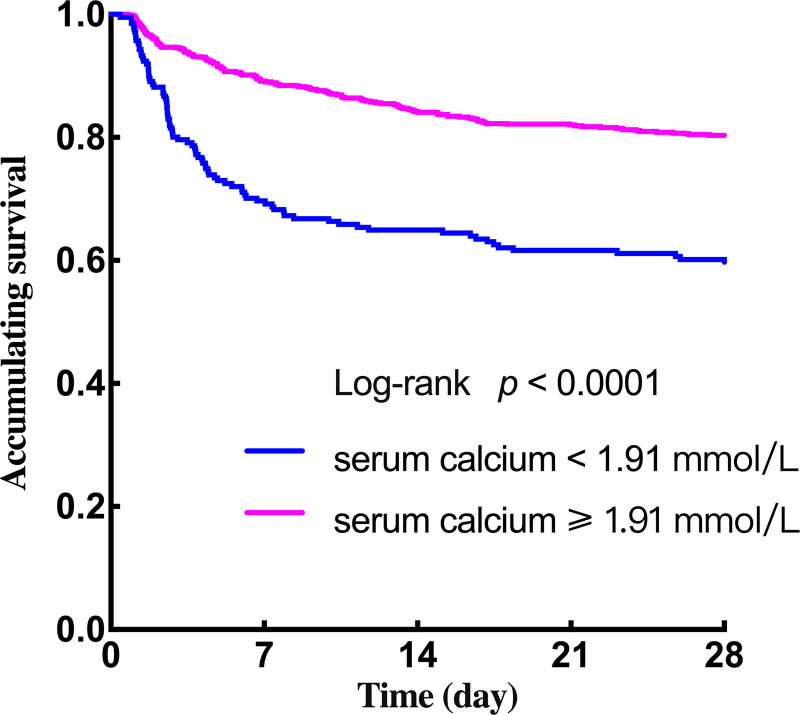
Kaplan–Meier curves of patients with low and high serum calcium levels.

Utilizing the Kaplan–Meier survival analysis, an evaluation was conducted to assess the association between serum calcium levels within 1 hour of ER admission and 28-day survival in sepsis. The result depicted in Figure 4 revealed a noteworthy trend that individuals with a lower serum calcium level (<1.91 mmol/L) exhibited a distinctly lower 28-day survival rate (*P* < .0001).

## 4. Discussion

In the present retrospective study, we aimed to investigate the potential association between serum calcium levels within 1 hour of ER admission and 28-day mortality in sepsis. Our findings shed light on the prognostic significance of serum calcium in sepsis, although the derived AUC from the ROC analysis demonstrated a relatively modest predictive capacity. This observation underscores the need to interpret the predictive value of serum calcium in conjunction with other established biomarkers and clinical indicators.

Our results revealed that lower serum calcium levels within 1 hour of ER admission were associated with a heightened risk of 28-day mortality among sepsis patients. Importantly, this association remains significant even after adjusting for various confounders such as age, CCI, vital signs, lab results, and APACHE II score. This is consistent with the prior studies. For instance, Li et al demonstrated that serum calcium levels are valuable in the early identification and assessment of disease severity in elderly patients with sepsis. Specifically, they found that sepsis patients with decreased serum calcium had higher shock rates and mortality rates.^[17]^ Similarly, Fei et al found an association between lower calcium levels and higher mortality rates in sepsis patients.^[18]^ Notably, the best cutoff value for predicting 28-day mortality risk was 1.92 mmol/L, mirroring our own conclusions. Such results reinforce the notion that serum calcium plays a role in sepsis outcomes. However, Hastbacka et al diverged from the consensus by suggesting that hypocalcemia did not independently predict 30-day mortality in critically ill adults.^[19]^ This discrepancy might be attributed to variations in patient characteristics, sample sizes, and study designs. While our study is based on a larger cohort, allowing for more robust evaluations, the conflicting findings highlight the intricate nature of sepsis and the need for comprehensive investigation.

Although the AUC for serum calcium alone was modest, its prognostic value is enhanced when considered alongside other variables such as CCI, APACHE II score, RDW, and Lac levels. The combination of these indicators substantially improves risk stratification, reflecting the multifactorial and interconnected pathophysiology of sepsis. Consistent with previous studies, our analysis emphasizes the prognostic significance of CCI,^[20,21]^ APACHE II score,^[22]^ RDW,^[23,24]^ and Lac levels.^[25]^ The CCI provides an overarching assessment of patient comorbidities, whereas APACHE II reflects organ dysfunction and overall disease severity. Elevated RDW and Lac levels, indicative of inflammation and tissue hypoperfusion, further enhance understanding of sepsis prognosis. Integrating these variables allows for a more precise and comprehensive assessment of patient risk.

Despite the prognostic importance of serum calcium, the clinical implications of calcium supplementation remain controversial. Melchers et al reported that parenteral calcium administration in critically ill patients with hypocalcemia did not improve outcomes and could even be harmful in certain subgroups.^[15]^ Fernandes and Pereira further emphasized that in critically ill patients, particularly those with sepsis, routine correction of moderate or mild hypocalcemia may not improve outcomes and can even increase mortality and organ dysfunction.^[26]^ These findings suggest that serum calcium levels should be interpreted cautiously, and supplementation should not be performed indiscriminately without considering patient-specific conditions.

A prominent strength of our study lies in its innovative approach to serum calcium measurement, conducted promptly upon ER admission, thus minimizing potential confounders associated with subsequent treatments. This methodological rigor enhances the credibility of our results and contributes to the field’s understanding of sepsis prognosis. Additionally, the utilization of a substantial sample size strengthens the robustness of our findings. However, our study has certain limitations that warrant consideration. The retrospective design introduces inherent biases, and future prospective studies are imperative to validate our results. Another constraint is the lack of data on PCT and CRP, pivotal sepsis biomarkers, because these markers weren’t routinely tested in our ER. Overlooking these markers might circumscribe the breadth of our prognostic model. Introducing a broader range of biomarkers and clinical indicators, encompassing PCT and CRP, might refine sepsis prognosis accuracy. Additionally, as a single-center study, the generalizability of our findings could be circumscribed. Despite these limitations, our study underscores the potential of serum calcium as an independent prognostic factor in sepsis, contributing valuable insights to the field and guiding future research endeavors.

## 5. Conclusion

In conclusion, our study further corroborates the association between serum calcium levels and sepsis prognosis. We identified low serum calcium level as a robust predictor of 28-day mortality among sepsis patients. This finding underscores the imperative for clinicians to incorporate serum calcium levels into their risk assessment. It is crucial to note that while serum calcium provides supplementary predictive value, it should be integrated with other prevailing biomarkers and clinical metrics for comprehensive assessment.

## Acknowledgments

I’m grateful to my colleagues for their support during this manuscript’s development. My sincere thanks to the peer reviewers and editors for their invaluable feedback.

## Author contributions

**Data curation:** Haoran Peng.

**Funding acquisition:** Lijie Qin, Yanwei Cheng.

**Software:** Haoran Peng.

**Supervision:** Yanwei Cheng.

**Validation:** Lijie Qin.

**Writing – original draft:** Lijun Xu.

**Writing – review & editing:** Lijie Qin, Yanwei Cheng.
